# Improvement of Storage Stability of Zein-Based Pickering Emulsions by the Combination of Konjac Glucomannan and L-Lysine

**DOI:** 10.3389/fnut.2022.955272

**Published:** 2022-07-11

**Authors:** Teng Song, Hui Liu, Abdul Razak Monto, Tong Shi, Li Yuan, Ruichang Gao

**Affiliations:** ^1^School of Food and Biological Engineering, Jiangsu University, Zhenjiang, China; ^2^College of Life Science, Anhui Normal University, Wuhu, China

**Keywords:** Pickering emulsion, L-lysine, zein, konjac glucomannan, synergistic effects

## Abstract

In this work, L-lysine (Lys) was employed together with konjac glucomannan (KGM) to fabricate zein colloidal particles (ZCPs) aimed at enhancing the storage stability of Pickering emulsions. With the addition of Lys, zein-Lys colloidal particles (ZLCPs) and zein-Lys-KGM (ZLKCPs) exhibited smaller particle size (133.64 ± 1.43, 162.54 ± 3.51 nm), polydispersity index (PDI) (0.10 ± 0.029, 0.13 ± 0.022), π value, and more adsorbed protein. Meanwhile, KGM underwent deamidation in an alkaline solution, so the emulsions stabilized by ZLKCPs exhibited a solid gel-like structure with higher storage modulus (G′) and loss modulus (G′′), leading to lower fluidity and better stability. The synergistic effects of Lys and KGM improved the stability of the emulsion. Hydrophobic interactions and hydrogen bonds were the main driving forces forming colloidal particles, which were determined by driving force analysis.

## Graphical Abstract

Zein self-assembled into colloidal particles with external hydrophobic ends and internal hydrophilic ends during the anti-solvent process; ZCPs and KGM are bonded to form a network structure in the form of hydrogen bonds; ZCPs and Lys are bonded by ion interaction and hydrogen bond and decrease the particle size of ZCPs; The synergistic effects of Lys and KGM improved the stability of the emulsion. 
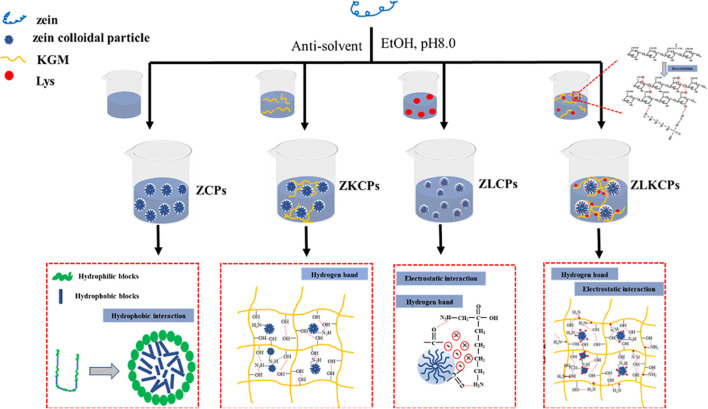

## Introduction

Due to its widespread use in foods, pharmaceuticals, and the cosmetic industry, there has been a steady interest in emulsion-based delivery systems in recent years. Pickering emulsions stabilized by solid particles with unusual surface activity have aroused the widespread interest of many scientists because of their numerous supplementary advantages, such as high interfacial stability, low toxicity, and low cost (1–3). Besides inorganic particles, bio-derived polymers are also used to stabilize Pickering emulsions, among which zein is a natural globular protein rich in hydrophobic leucine, alanine, and proline acids, which has been used as a stabilizer of Pickering emulsions (4) due to its amphiphilic nature, self-assembly characteristics, good biocompatibility, and adhesion properties (5). However, for over 50% of amino acid residues on the surface of zein with poor stability at the iso-electric point (around pH 6), Pickering emulsions stabilized merely by zein are prone to delamination, demulsification, oil leakage, and so on. These phenomena limit the utilization of zein in many commercial applications. As a result, several approaches have been developed to make surface modifications of zein.

In recent years, many scientists have been devoted to studying the synergistic effects of zein and other substances on the stability of emulsions, in which protein-polysaccharide complexes formed via electrostatic interaction and increasing the viscosity can be used for emulsion stabilization (6–8). As a hydrophilic dietary fiber, konjac glucomannan (KGM) is derived from tubers of konjac and was reported to possess many health benefits, such as lowering blood sugar and blood cholesterol levels, improving the intestinal microenvironment and immune function, promoting weight loss, and preventing fatty liver (9–11). Due to its hydrophilic, gelating, and thickening properties, KGM has been widely used in food, pharmaceutical, and other fields. It is reported that KGM can improve the emulsions’ creaming, pH stability, and protect them from oiling-off after the freeze-thaw process (9). Moreover, it has been reported that KGM deacetylated in alkaline solutions, forming more homogeneous junction points and more elastic gels network (12), in which the oil drop was fixed firmly. Therefore, using KGM to improve the stability of zein-based emulsions in alkaline solution is workable.

Naturally occurring amino acids including arginine (Arg), glycine (Gly), proline (Pro), histidine (His), alanine (Ala), and glutamic acid (Glu) have been broadly used as additives to improve protein solubility by suppressing aggregation in the field of food and medicine (5, 13, 14). Among these novel amino acid additives, particularly essential amino acids such as L-lysine (Lys) and Arg have been demonstrated to stabilize protein emulsions under stress conditions and improve protein water holding capacity, viscoelasticity, texture profile, and sensory attributes of the protein gel. Studies showed that Lys could hinder myosin aggregation and increase the solubility of myosin (13). Furthermore, it has been found that Lys could markedly improve the emulsion stability and cooking loss of emulsion sausages with soybean oil, which was associated with the increase in electrostatic repulsion of emulsion droplets and the decrease in oil-water interfacial tension by adding Arg and Lys (15).

It is worth mentioning that the Lys solution is alkaline. Would KGM undergo deamidation in Lys’ solution and further improve the properties of the emulsion gel? What are the key factors affecting the synergism? What is the mechanism by which Lys and KGM act synergistically?

In this study, zein, Lys, and KGM were used to prepare colloidal particles and the effects of reaction sequence and time were studied. In addition, the properties of emulsions stabilized by these colloidal particles were further evaluated.

## Materials and Methods

### Materials

Zein (protein content of 90%) was purchased from Sigma-Aldrich, Steinheim, Germany. Konjac glucomannan (KGM) was supplied by Zhengya Chemical Products Co., Ltd. (Zhengzhou, China). Fish oil was self-made using papain and neutral protease and the final extraction rate was 85.26%. All other chemicals were of analytical grade and purchased from Sinopharm Chemical Reagent Co., Ltd. (Shanghai, China).

### Preparation of Zein-Lys-Konjac Glucomannan Colloidal Particle

#### Preparation of Zein Colloidal Particles

Prepare 4.0% (w/v) zein solution by dissolving zein in 75% (v/v) ethanol solution. Then, zein was fully dispersed in solution by continuous stirring for 1 h with a magnetic stirrer (C-Mag HS 7 control, IKA, Germany) at room temperature. The pH was adjusted to 8.0 using 0.1 M NaOH or HCl. Then, the zein solution was dribbled into 300 mL of distilled water drop by drop and magnetically stirred for 1 h. Subsequently, ZCPs dispersion, with the zein concentration of 4.0% (w/v), was obtained by rotary evaporation. Finally, the prepared ZCPs dispersion was centrifuged at 3,000 rpm for 5 min to remove large particles and aggregates. Some samples were stored at 4°C and the remainders were freeze-dried for further analysis.

#### Preparation of Composite Colloidal Particles and Pickering Emulsion*s*

Lys was dissolved in distilled water to prepare a 0.4% Lys aqueous solution (w/v). The pH was adjusted to 8.0 using 0.1 M NaOH or HCl. Then, ZCPs dispersion was added to an equal volume of Lys aqueous solution and thoroughly mixed using a magnetic stirrer. Finally, zein-Lys composite colloidal particle (ZLCPs) dispersion was obtained.

Konjac glucomannan powders were added to the Lys aqueous solution (pH = 8.0) at 50°C, configuring the KGM concentration of 0.4% (w/v). Moreover, Lys-KGM solution was obtained. Subsequently, an equal volume of the ZCPs dispersion was added to the Lys-KGM solution at 700 rpm to obtain zein-Lys-KGM composite colloidal particles (ZLKCPs) dispersion.

The oil phase was added to the aqueous phase dispersion and the volume fraction of the oil phase was 50%. Pickering emulsions were prepared by homogenizing the mixtures using an Ultra-Turrax T18 (IKA, Germany) homogenizer at a constant speed of 11,000 rpm for 2 min. The emulsions were transferred into glass bottles and stored at 4°C for further studies.

#### The Effect of Reaction Sequence and Time of Zein, L-Lysine, and Konjac Glucomannan on the Appearance and Stability of the Emulsion

Different types of colloidal particles were prepared: (i) Zein-Lys-KGM ternary composite colloidal particles were prepared after 0 h reaction time; (ii) Zein-KGM composite colloidal particles pre-mixed for 1, 3, 6, 12, and 24 h, and then the suspension was mixed with Lys solution; (iii) Zein-Lys composite colloidal particles were mixed for 1, 3, 6, 12, and 24 h in advance and then mixed with KGM solution; (iv) The Lys-KGM mixed solution was pre-mixed for 1, 3, 6, 12, and 24 h and then mixed with zein nanoparticle dispersion to prepare an emulsion.

### Characteristics of Colloidal Particles

#### Particle Size, Polydispersity Index, and Zeta Potential of the Colloidal Particles

The Mastersizer 3000 laser particle size analyzer was used to determine the colloidal particles’ particle size, PDI, and zeta-potential (Malvern Instruments, Worcestershire, United Kingdom). In brief, the colloidal suspension was diluted 100 times; about 600 μL of the samples were placed in cuvettes and the particle size was determined according to a previous study by Zou et al. (16) with some modifications. The measurement temperature was set at 25°C, and all operations were repeated three times.

#### Morphological Characteristics of Colloidal Particles

According to a reported study (17), the morphology of the colloidal particles was observed using a Hitachi S-4700 (Hitachi, Tokyo, Japan) scanning electron microscopy (SEM). In brief, freeze-dried samples were fixed on the conductive carbon tape and sprayed with a gold layer to avoid electrostatic effects. Images of all samples were collected with a field emission scanning electron microscopy (Quanta FEG 250, FEI Company, Hillsboro, OR, United States) with an accelerating voltage of 20.0 kV.

#### Fourier Transform Infrared Spectroscopy

The secondary structure of freeze-dried samples was determined according to a previous method (18). An Fourier transform infrared spectroscopy (FTIR) spectrometer (Nicolet iS10, Thermo-Scientific, Jasco Inc., Easton, MO, United States) was used to investigate the changes in zein, Lys, and KGM. Each sample (6.0 mg) was mixed with KBr (194 mg) and the spectrum range was from 4,000 to 400 cm^–1^ in 32 scans with a 4 cm^–1^ resolution.

#### Circular Dichroism Measurement

The change in the secondary structure of zein with the addition of Lys and KGM was investigated using a circular dichroism (CD) spectropolarimeter (Pistarp-180, Applied Photophysics Ltd., Surrey, London, United Kingdom). First, the colloidal particle suspensions were diluted to 0.05 mg/mL and the sample diluents were filtered with 0.22 μm syringe filters. A sample container with an optical path length of 1 cm was used and the wavelength ranged from 190 to 250 nm. The experiment was conducted at 25°C under nitrogen and repeated three times, and the average value was taken. CDPro was used to calculate the secondary structure.

#### Fluorescence Spectroscopy and Surface Hydrophobicity

According to a previous method (19, 20), a fluorescence spectrophotometer (Model Cary Eclipse, Varian Inc., Palo Alto, CA, United States) was used to determine fluorescence spectroscopy and surface hydrophobicity. For fluorescence spectroscopy, the emission wavelength ranged from 290 to 450 nm and the excitation wavelength was 280 nm (slit width = 5 nm). Furthermore, for surface hydrophobicity, a fluorescent probe named 8-8′-Diphenyl-5,5′-dinaphthalene disulfonate (ANS) was used to study the structure and behavior of zein. In brief, colloidal particle suspensions were diluted with distilled water to different concentration gradients. Then, the diluted protein suspensions with the volumes of 4 mL and 20 μL of 8 mM ANS were mixed to aid in analyzing surface hydrophobicity. Subsequently, 470 and 390 nm emission wavelength and excitation wavelength, respectively, were selected for the measurement of fluorescence intensity. The slope of the linear plot of the net fluorescence values versus protein concentrations was used to index the protein surface hydrophobicity.

#### Characterization of Interfacial Properties

The ratio of adsorbed protein (AP) onto the O-W interface of the fresh emulsion was determined according to Taha et al. (21) with some modifications. About 1 mL of emulsions were centrifuged at 4°C at 12,000 × g for 35 min for phase separation. The serum phase (Cs) was carefully collected and filtered through a 0.22-μm syringe filter to remove impurities. The protein concentration of the Cs was measured with the Kjeldahl method. The AP (%) was calculated using the following equation [Eq. (1)]:


(1) $$AP=\frac{C_{0}-C_{s}}{C_{0}}\times 100$$

where *C*_0_ refers to the emulsion’s total protein concentration (mg/mL) and *C*_*s*_ refers to the protein concentration of the continuous serum phase.

According to a previous study (22), an Optical Contact Angle Measuring Device (OCA20, Dataphysics Company, Germany) was used to determine the adsorption kinetics of colloidal particles at the O-W interface. The detailed procedures were as follows: first, colloidal particle suspension was loaded into a syringe with a concentration of 0.1 mg/mL; at the same time, the soybean oil was loaded into a U-shaped test cell; then, the plunger of the syringe was pushed and a 10-μL oil drop was formed at the tip of the syringe. The interfacial tension was continuously monitored. The process of adsorption was measured by recording the interfacial pressure π, which was calculated by the following equation [Eq. (2)]:


(2) $$\pi =\sigma_{0}-\sigma$$

where σ represents the interfacial tension of the colloidal particle suspensions, σ_0_ represents the interfacial tension of the pure water, and σ_0_ = 72.0 mN/m.

#### Driving Force Analysis

Reactive additives, such as urea and SDS, were used to analyze the driving force among zein, Lys, and KGM (23). In short, 0–3 M urea and 0.05–0.1% (w/v) SDS were added to the colloidal particle suspension. Turbidity was measured after reacting for 15 min. The variation in turbidity indicated the effects of different non-covalent bonds in the complex.

### Characteristics of Pickering Emulsions

#### Droplet Size of Pickering Emulsions

The droplet size of Pickering emulsions was measured using a Mastersizer3000 laser particle size analyzer (Malvern Instruments, Worcestershire, United Kingdom) as described in section “Particle Size, Polydispersity Index, and Zeta-Potential of the Colloidal Particles”. Furthermore, the volume fraction-length (D4,3) and volume-area (D3,2) average diameters were calculated using Eqs (3, 4):


(3) $$D_{4,3}=\frac{\sum n_{i}d_{i}^{4}}{\sum n_{i}d_{i}^{3}}$$

(4) $$D_{3,2}=\frac{\sum n_{i}d_{i}^{3}}{\sum n_{i}d_{i}^{2}}$$

Where *n*_i_ is the number of particles with a diameter of *d*_i_.

#### Rheological Properties of Emulsions

The rheological properties of emulsions were determined using a rotational rheometer (TA Instruments, New Castle, DE, United States), which was scanned at 25°C according to Zhou et al. (24) with slight modifications. In brief, emulsions were deposited on a 40-mm plate for 5 min to allow temperature equilibration before each measurement. For the steady shear flow tests, the shear rate was increased from 0.1 to 100 s^–1^ to obtain the apparent viscosity. For the dynamic measurements, frequency sweeps were carried out by increasing the frequency from 0.1 to 100 Hz with a strain of 0.5%, which was within the linear viscoelastic range. All rheological measurements were performed at least three times.

#### Confocal Laser Scanning Microscopy Analysis

A Leica TCS SP8 confocal microscope (Leica Microsystems Inc., Heidelberg, Germany) was used for laser confocal analysis referring to a previous study (25) with slight modifications. In brief, 20 μL of 0.1% (w/v) Nile Red and 10 mg/mL Fast Green were added to 1 mL of emulsions and stained for 1 h. Then, 10 μL of stained samples were placed on glass slides, covered with a cover glass, and placed upside down on a loading table for observation. The fluorescent dyes were excited at 488 nm for Nile Red and 633 nm for Fast Green.

### Statistical Analysis

The data were processed and reported as the mean ± standard deviation. One-way analysis of variance (ANOVA) was performed at the probability level of 0.05 according to the *post hoc* Tukey’s test. Statistical analysis was conducted using Origin 2018 (OriginLab Co., Northampton, MA, United States).

## Results and Discussion

### The Effect of Reaction Sequence and Reaction Time on the Appearance and Stability of Emulsions

**FIGURE 1:**
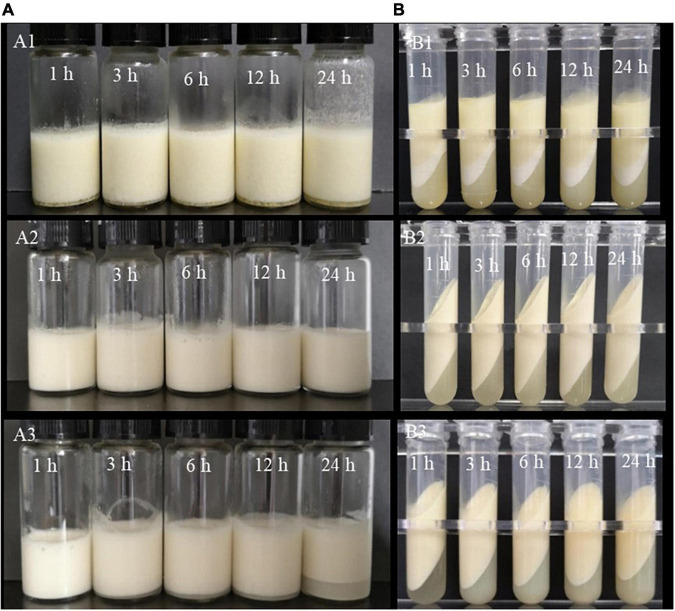
Morphology **(A1–A3)** and centrifugal stability **(B1–B3)** of Pickering emulsions stabilized by ZKCPs, ZLCPs, and ZLKCPs; 1, 3, 6, 12, and 24 h stood for the pre-mixing time.

O-W emulsions were successfully prepared as soon as zein (Supplementary Figure 2), Lys, and KGM were mixed, but the emulsions stratified and a serum layer appeared in the lower layer after being stored for 2 days (Figures 1A,B and Supplementary Figure 1).

Zein colloidal particle suspension was mixed with KGM and pre-reacted for a gradient time, then Lys was added to the mixer and incubated for 1 h. Subsequently, emulsions were prepared with the suspension. As shown in Figure 1A1, the emulsions showed rough textures and serum-like layers at the bottom after being stored for 2 days. Furthermore, flocculation appeared on the vial’s wall and the emulsions’ bottom. After centrifugation, more composite particles were at the bottom due to flocculation and aggregation (Figure 1B1).

Lys solution was added to the same volume of zein colloidal particle suspension and pre-reacted for gradient time (1, 3, 6, 12, and 24 h), then KGM was added and incubated for 1 h. As shown in Figure 1A2, all emulsions did not delaminate after being stored at room temperature for 2 days, and there was no oil phase precipitation after centrifugation (Figure 1B2), exhibiting better centrifugal stability. The appearance of emulsions did not change with the extension of pre-reaction time, indicating that it took a short time to complete the reaction between ZCPs and Lys.

As shown in Figure 1A3, no stratification was observed for the emulsions prepared by colloidal particles with Lys and KGM pre-acting for 1 h after being stored for 2 days. The emulsions, prepared by colloidal particles with Lys and KGM pre-reacting for 3 h, stratified after being stored for 2 days, and the lower water layer increased as the pre-mixing time was prolonged. The water layer at the bottom of the emulsion, which was prepared with Lys-KGM pre-mixing for 24 h, increased significantly.

### Characteristics of Colloidal Particles

#### Particle Size, Polydispersity Index, and Zeta Potential of the Colloidal Particles

**FIGURE 2:**
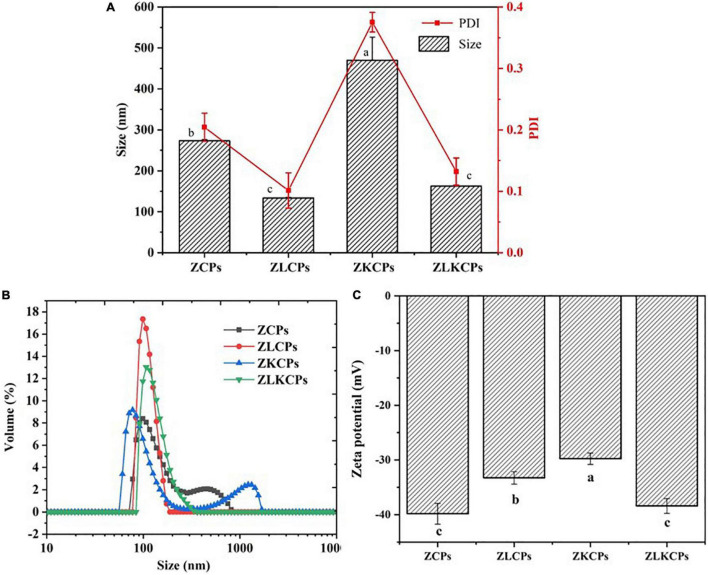
Average diameter and polydispersity index (PDI) **(A)**, the distribution of particle size **(B)**, and the zeta potential **(C)** of different colloidal particles.

As shown in Figures 2A,B, the average diameter size distribution of ZCPs, ZLCPs, ZKCPs, and ZLKCPs was determined. The average diameter of ZCPs was 273.19 ± 3.27 nm at pH 8.0, and the particle size distribution exhibited a bimodal shape, including one peak distributed around 100 nm and the other peak appeared in the range of 400–500 nm. The particle size of ZKCPs also showed a bimodal distribution, with the prominent peak distributed around 80 nm and a peak distributed around 1,300 nm, with an average particle size of 469.94 ± 56.49 nm. During the anti-solvent process, the zein molecules aggregated and formed colloidal particles with larger particle sizes. With the addition of KGM, the particle size of the colloidal particles increased. Deamidation of KGM occurred because of the alkaline environment, causing the association between acetyl-free regions of the backbone, leading to the formation of junction zones and an increase in particle size. However, adding significantly reduced the particle size of zein nanoparticles (133.64 ± 1.43 nm). Lys could inhibit zein’s aggregation behavior, change the solution’s pH value, and occupy hydrophobic binding sites, thereby reducing the particle size (26). The zeta potential of ZCPs, ZLCPs, ZKCPs, and ZLKCPs were −39.82 ± 1.893, −33.25 ± 1.131, −29.76 ± 1.051, and −38.39 ± 1.348 mV, respectively (Figure 2C). The addition of Lys neutralized the negative charge of the ZCPs suspension. Simultaneously, KGM wrapped around the colloidal particles and also covered part of the charge, resulting in a decrease in negative charge.

#### Morphological Characteristics of the Colloidal Particles

**FIGURE 3:**
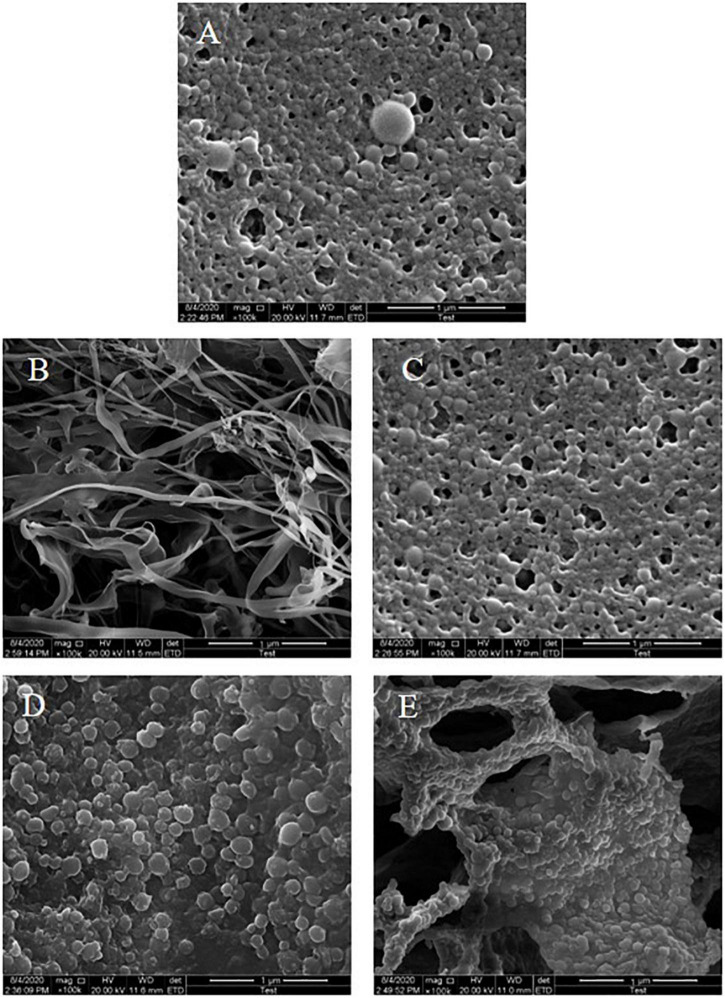
Scanning electron microscopy (SEM) images of composite colloidal particles (**A:** ZCPs, **B:** KGM, **C:** ZLCPs, **D:** ZKCPs, and **E:** ZLKCPs).

The microstructure of freeze-dried samples of different colloidal particles was observed using SEM with a magnification of 100,000 times. As shown in Figure 3A, a zein colloidal particle possessed a typical spherical structure and uneven particle size distribution at pH 8.0 due to aggregation of zein during the antisolvent process. The KGM power was fibrous and cross-linked to form a network-like structure (Figure 3B). For ZLCPs (Figure 3C), the particles were smaller and more uniform with the addition of Lys, indicating that the addition of Lys changed the aggregation state of zein colloidal particles (27). ZKCPs particles (Figure 3D) showed no singly spherical or fibrous structure but rough branch shapes with spherical particles tightly adsorbed on the surface of the “branch,” and the spherical structure of the particles exhibited no irregularities when aggregation occurred. For ZLKCPs (Figure 3E), the spherical particles were still tightly covered on the surface and attached to the “branches,” which were more regular and uniformly distributed. Compared to KGM power, the “branch” seemed to be broken into a finer and mesh-like structure.

#### Fourier Transform Infrared Spectroscopy Analysis

**FIGURE 4:**
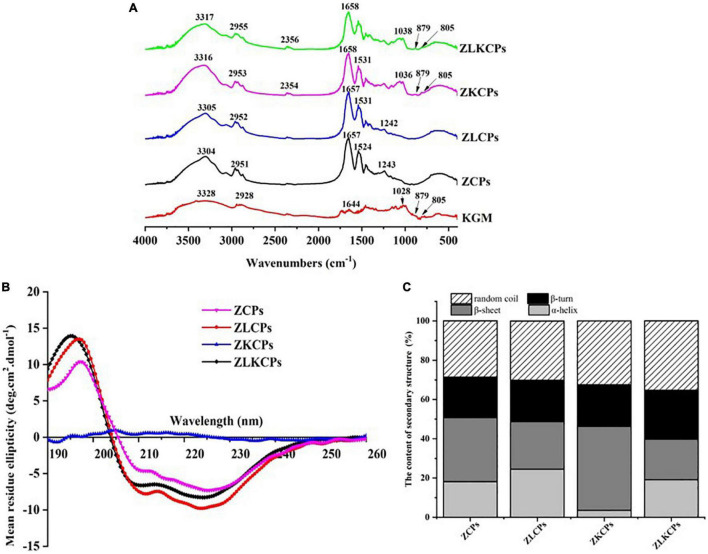
Fourier transform infrared spectroscopy spectra **(A)**, circular dichroism (CD) spectroscopy spectra **(B)**, and the content of secondary structure **(C)**.

The FTIR spectrum of KGM, ZCPs, ZLCPs, ZKCPs, and ZLKCPs was obtained in the wavelength region of 4,000–400 cm^–1^ (Figure 4A). ZCPs and KGM exhibited their characteristic peaks at 3,304 and 3,328 cm^–1^, respectively, caused by the stretching vibration of –OH (28). With the addition of L-Lys and KGM, the peaks shifted to 3,305 cm^–1^ (ZLCPs), 3,316 cm^–1^ (ZKCPs), and 3,317 cm^–1^ (ZLKCPs), respectively, indicating that the hydrogen bond (3,200–3,400 cm^–1^, N–H stretching) might form among zein, Lys, and KGM (29). ZCPs and KGM peaks occurred at 2,951 and 2,928 cm^–1^ due to the stretching vibration of hydrophobic C–H (30), which were also observed. Since there is a high proportion of hydrophobic amino acids and the deacetylation of KGM, there should be a hydrophobic interaction between ZCPs and KGM. Moreover, the peak shapes and intensity of ZLCPs, ZKCPs, and ZLKCPs changed slightly with the addition of Lys and KGM, which might have reduced the hydrophobic stretch band of ZCPs, indicating that hydrophobic interaction was involved in the formation of composite nanoparticles.

The peaks of KGM at 1,644 and 1 028 cm^–1^ were obtained, which were contributed by intramolecular hydrogen bonding, C–O stretching vibration, and mannose unit stretching vibration, respectively (31). Meanwhile, two characteristic absorption peaks were observed near 805 and 879 nm^–1^ due to the basic unit of the mannan in KGM, which can be attributed to the presence of a D-mannose residue in the polymers irrespective of the configuration and the position of the mannosidic linkage. These two absorption pecks were also observed for ZKCPs and ZLKCPs, indicating that the backbones of KGM samples were the same. Furthermore, the peaks of ZCPs observed at 1,657 and 1,524 cm^–1^ were contributed by the amide I band (1,750–1,600 cm^–1^) and amide II bands (1,550–1,510 cm^–1^) (32), respectively. The amide I band was mainly related to C=O stretching vibration and amide II band was mainly related to N–H bending and C–N stretching vibration.

The peak of KGM at 1,028 cm^–1^ was not observed in the spectrum of ZKCPs and ZLKCPs, but there were peaks at the wavenumber of 1,036 and 1,038 cm^–1^, which might be ascribed to C–O stretching vibration (1,300–750 cm^–1^) (33), which the acetyl group might have induced in KGM when it interacted with the amide group in zein. Furthermore, new peaks were observed at 2,354 and 2,356 cm^–1^, which might be the cumulative asymmetric stretching motion of the double bond caused by the interaction of zein, L-Lys, and KGM (34). FTIR results indicated intermolecular interactions between zein, Lys, and KGM, not only electrostatic interactions but also hydrogen bonding and hydrophobic interactions.

#### Circular Dichroism Analysis

Circular dichroism spectroscopy is one of the most effective technical methods to investigate protein secondary structure. Figure 4B showed the difference in the far ultraviolet CD spectrum of zein colloidal particles in the presence of Lys and KGM. The typical α-helix-rich structure of ZCPs had a positive absorption peak around 193 nm, two negative absorption peaks at 209 and 229 nm, and a zero-crossing point around 203 nm. The curvature of the spectral curve of the composite colloidal particles at 209 and 223 nm changed significantly (*p* < 0.05), indicating that the addition of Lys and KGM changed the secondary structure of ZCPs. It has been reported that the content of the α-helical structure of native zein ranged from 30 to 60%, and most of the remaining structures were random coils (35). Typically, α-helix reflects the order of protein molecules, while β-sheets, β-turns, and random coils reflect the looseness of protein molecules (36). The secondary structure composition of ZCPs was 18.1% α-helix, 32.0% β-sheet, 20.7% β-turn, and 29.2% random coils at pH 8.0 (Figure 4C). With the addition of KGM, the α-helix of ZKCPs reduced to 3.5%, but β-sheets and random coils increased to 42.7 and 32.4%, respectively. It was found that more β-sheets are usually found in aggregated proteins (36), which confirmed that at pH 8.0, ZCPs and ZKCPs aggregated, flocculated, and precipitated, so the emulsification ability was reduced. With the addition of Lys, the α-helix of ZLCPs and ZLKCPs increased to 25.8 and 19.1%, while the β-sheets reduced to 20.3 and 20.6%, the random coil increased to 32.8 and 35.4%, respectively, indicating that the addition of Lys inhibited the aggregation of ZCPs and ZKCPs, restoring the secondary structure of ZCPs to an orderly initial structure, and also promoted changes in the spatial structure of zein molecules to stretch fully.

#### Fluorescence Spectroscopy and Surface Hydrophobicity

**FIGURE 5:**
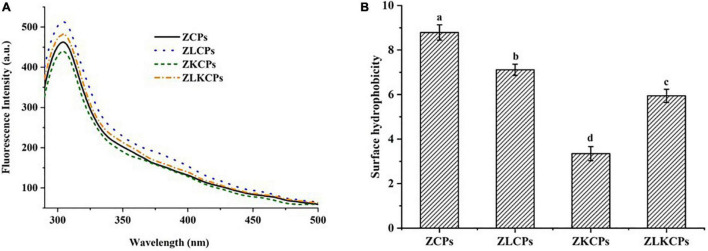
Fluorescence spectroscopy spectra **(A)** and surface hydrophobicity **(B)**.

The effects of Lys and KGM on the fluorescence properties of zein were shown in Figure 5A. The ZCPs showed the maximum emission peak of the fluorescence spectrum at 304 nm, which was consistent with a previous report (37). With the addition of KGM, the maximum emission wavelength of ZKCPs remained invariable, but the fluorescence intensity decreased. Protein aggregation resulted in the shielding of tryptophan (Trp) residues, while the unraveling of protein molecules led to the exposure of Trp residues, which were usually buried in the hydrophobic core of proteins. The Trp residues were buried due to the aggregation of ZKCPs, resulting in the reduction of the fluorescence intensity. The fluorescence intensity of composite colloidal particles was enhanced by adding Lys. It might be attributed to the fact that Lys induced the melting of the zein molecular chain, prevented the aggregation of ZCPs, relaxed the zein molecule’s conformation, and exposed more Trp residues, increasing fluorescence intensity. In addition, compared with ZLCPs, the fluorescence intensity of ZLKCPs decreased. It could be explained that ZLCPs combined with KGM and formed a particle-filled micro-gel structure, which could be confirmed by SEM results.

To further explain the interaction between zein, Lys, and KGM, ANS was used as a fluorescent probe to detect the surface hydrophobicity of the protein (Figure 5B). More than 50% of the amino acids in native zein were hydrophobic resulting in a strong surface hydrophobicity at pH 8.0 (38). The surface hydrophobicity of ZCPs reduced with the addition of KGM, contributing to ZKCPs flocculating and precipitating at pH 8.0, which reduced the solubility and the ability to bind to ANS, resulting in lower surface hydrophobicity. ZLCPs and ZLKCPs exhibited lower hydrophilicity and smaller particle size, which was more conducive to the colloidal particles densely adsorbed at the oil-water interface, thereby enhancing the emulsifying ability, which also provided a theoretical basis for the successful preparation of emulsion with the addition of Lys.

#### Characterization of Interfacial Properties

**FIGURE 6:**
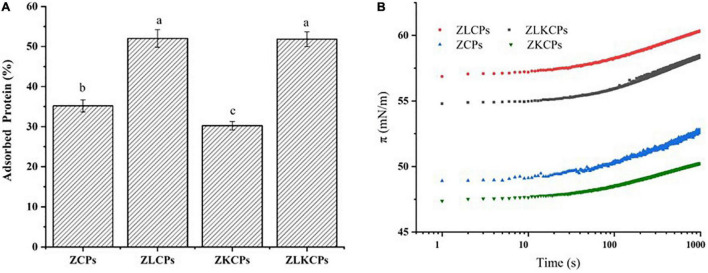
The adsorbed protein **(A)** and interfacial tension **(B)** of different samples.

As an emulsifier, colloidal particles adsorb on the surface of the oil drop, leaving the hydrophilic sides in contact within the aqueous phase (39). As shown in Figure 6A, ZLCPs and ZLKCPs exhibited the highest surface-adsorbed proteins, 52.02 ± 2.02 and 51.83 ± 1.86%, respectively. Followed by ZCPs and ZKCPs, 35.17 ± 1.53 and 30.26 ± 1.07%, respectively, indicating that the addition of Lys could increase the amount of adsorbed proteins. Notably, the change of adsorbed protein was consistent with that of particle size, which was also confirmed in the study by Shi et al. (39). The separation of colloidal particles from the interface needs to break through a vast reverse desorption energy barrier, which makes the adsorption irreversible and endows Pickering emulsion with high stability (40). For a single spherical particle, the desorption energy is related to the particle size, three-phase contact angle, and the oil-water interfacial tension. The smaller the radius, the larger the energy barrier to be overcome for the desorption, and the more difficult it was for the protein to be resolved from the oil-water interface, thereby providing steric hindrance to prevent the emulsion droplets from aggregating.

In the two-phase system, the phase interface is broken as the emulsification process proceeds, and the solid particle emulsifier rapidly adsorbs on the new oil-water interface, reducing the interfacial tension and forming a physical barrier preventing aggregation between droplets, so interfacial tension is a measure of the particle’s ability to emulsify. As shown in Figure 6B, ZCPs, ZLCPs, ZKCPs, and ZLKCPs could significantly reduce the interfacial tension of water. ZCPs and ZKCPs showed higher π values than ZLCPs and ZLKCPs, indicating that Lys was favorable for the rapid adsorption of colloidal particles at the O-W interface.

#### Driving Force Analysis

**FIGURE 7:**
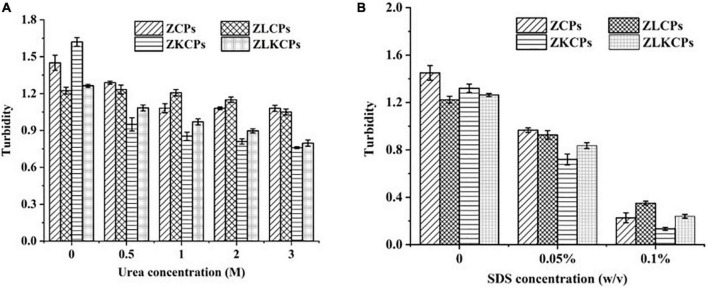
Effects of different dispersants on colloidal particles: **(A)** Urea; **(B)** SDS.

The main driving forces for forming complex colloidal particles are non-covalent interactions, such as hydrophobic and hydrogen bonding interactions. Different denaturing reagents (urea for hydrogen bonds and sodium dodecyl sulfate (SDS) for hydrophobic interactions) were used to assess the interactive forces between the colloidal particles. As shown in Figure 7A, the turbidity of ZCPs was 1.45 (Urea concentration was 0%), which was higher than that of ZKCPs (1.22), indicating that KGM combined with ZCPs by hydrogen bonds. Meanwhile, the turbidity of ZLKCPs was 1.76 ± 0.012, which was the highest among all the samples. It was ascribed to the fact that the hydroxyl groups of the KGM sugar chain formed intramolecular and intermolecular hydrogen bonds after deacetylation. With the addition of urea, the turbidity of all samples decreased. The higher the concentration of urea, the more turbidity decreased. When the concentration of urea was 3 M, the turbidity of the samples was the lowest, which were 1.08, 1.05, 0.76, and 0.79, decreasing by 25.52, 14.17, 53.09, and 55.79%, respectively. Compared with ZCPs and ZLCPs, the turbidity changes of ZKCPs and ZLKCPs were even more significant, indicating that hydrogen bonds played greater roles in forming ZKCPs and ZLKCPs, and KGM combined with zein mainly by hydrogen bonds. Compared with ZCPs, the turbidity of ZLCPS decreased less sharply, indicating that the addition of Lys disrupted hydrogen bonds to a certain extent. For ZLKCPs, turbidity was reduced by 55.79%, indicating that the number of hydrogen bonds broken by Lys is roughly equal to the number of new ones formed.

Sodium dodecyl sulfate was used to explore the possibility of hydrophobic interactions (41). SDS significantly reduced the turbidity of the ZCPs dispersion (Figure 7B). As the concentration of SDS increased, the turbidity continued to decrease. When the concentration of SDS was 0.1%, the turbidity of the samples were 0.23, 0.35, 0.13, and 0.24, reducing by 84.36, 71.39, 89.90, and 81.00%, respectively, which indicated that hydrophobic interaction was the main driving force during the formation of the colloidal particles. Comparing the turbidity of ZCPs and ZKCPs with those of ZLCPs and ZLKCPs indicated that the hydrophobic interaction in the colloidal particles was weakened with the addition of L-Lys confirmed by Gao et al. (42).

### Characteristics of Pickering Emulsions

#### Droplet Size of Pickering Emulsions

**FIGURE 8:**
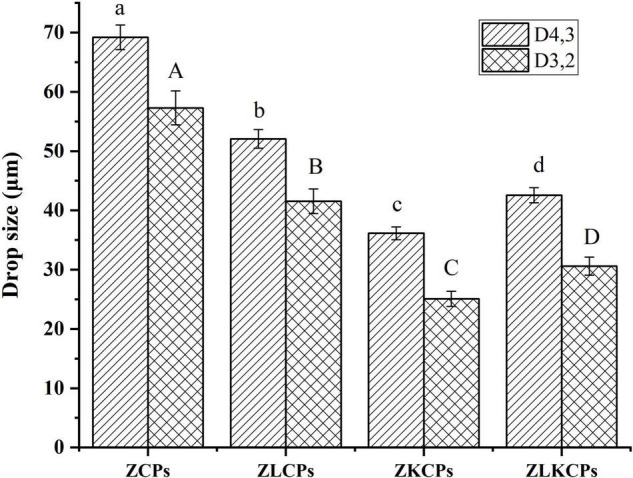
Droplet size of Pickering emulsions.

**FIGURE 9:**
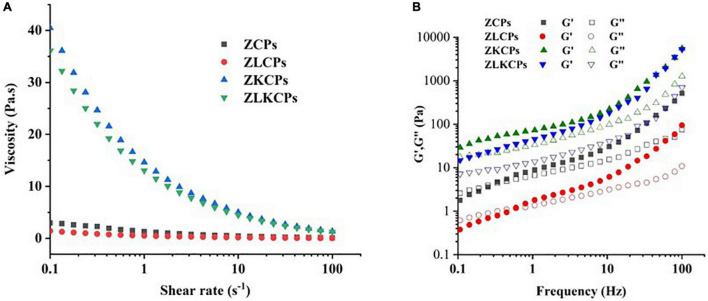
Rheological properties of Pickering emulsion stabilized by different colloidal particles [**(A)** viscosity; **(B)** Storage modulus (G′) and loss modulus (G′)].

The mean droplet sizes (D_4_,_3_ and D_3_,_2_) of Pickering emulsions stabilized by ZCPs, ZLCPs, ZKCPs, and ZLKCPs were shown in Figure 8. It can be found that all mean droplet sizes (D_4_,_3_ and D_3_,_2_) of Pickering emulsions were micrometer scale. The emulsion stabilized by ZCPs showed the largest particle size [D_4_,_3_ = 69.21 ± 2.08, D_3_,_2_ = 57.28 ± 2.86 μm) with an irregular shape (Figures 9A1–A3). It is possible that the emulsion stabilized by ZCPs was unstable, and the oil droplets aggregated, thereby resulting in the larger particle size of the emulsions. With the addition of Lys, the particle size of the emulsion stabilized by ZLCPs exhibited a regular shape with a smaller particle size (D_4_,_3_ = 52.07 ± 1.56 μm, D_3_,_2_ = 41.56 ± 2.08 μm) since Lys could inhibit the aggregation of oil droplets. The addition of KGM increased the particle size of the emulsions compared with those stabilized by ZLCPs, and the emulsions stabilized by ZLKCPs exhibited the smallest particle size due to the synergy of Lys and KGM, which was consistent with the results of CLSM.

#### Rheological Properties of Emulsions

The structure of emulsions by rheology is of great significance for the design and application of Pickering emulsion systems in the food industry (5). To better study the effects of Lys and KGM on emulsion stability, the response of shear rate to emulsion viscosity and frequency to emulsion viscoelasticity were determined. As shown in Figure 9A, the viscosity of emulsions significantly decreased with an increasing shear rate, indicating a shear-thinning property. Furthermore, KGM combined with ZCPs formed a 3-dimensional (3D) network structure during the emulsification, improving the emulsion system’s viscosity, increasing steric hindrance, and relieving the shear thinning behavior (43). The viscosity of the emulsions stabilized by ZCPs and ZKCPs was higher than that of the emulsions stabilized by ZLCPs and ZLKCPs, which could be ascribed to the Lys disturbing the hydrophobic interaction between colloidal particles, thereby reducing the viscosity of the emulsions. Furthermore, the deacetylated KGM varied from the semiflexible straight-chain into an elastic microsphere, resulting in asymmetry and ordered structure, thereby decreasing the viscosity of the emulsions stabilized by ZLKCPs.

Figure 9B revealed the frequency dependence of the elastic modulus (G′) and viscoelastic modulus (G′′) of different emulsions in the dynamic frequency sweep range of 0.1–100 Hz. It was found that the values of G′ and G′′ were affected by Lys and KGM. For the emulsions stabilized by ZCPs and ZLCPs, the G′′ was superior to G′ at a frequency range of 0.1 and 0.6 Hz, and then the growth rate of G′ was greater than that of G′′, thereby leading to G′ being superior to the frequency range of 0.6–100 Hz, indicating that emulsions exhibited liquid-like characteristics. In addition, the G′ and G′′ of the emulsions stabilized by ZCPs were superior to those stabilized by ZLCPs, indicating that the introduction of Lys weakened the emulsions’ solid-like behaviors (44). With the addition of KGM, G′ and G′′ increased significantly and the G′ was superior to G′′ in the entire frequency range, which showed that KGM strengthened the emulsions’ solid-like behaviors for emulsion gels to be formed. Simultaneously, the G′ and G′′ of the emulsions stabilized by ZKCPs were more remarkable than that of the emulsion stabilized by ZLKCPs. The result was consistent with the changes in ZCPS and ZLCPS. Zein structural unfolding converted the protein backbones to a relatively static unfolded state. Lys, with an opposite charge of zein at pH 8.0, approached the zein by electrostatic interactions and disturbed hydrophobic interactions between colloidal particles, which led to stiffer zein backbones (45). However, for emulsions stabilized by ZLKCPs, the deacetylated KGM exhibited higher water absorbency and more excellent intermolecular interactions with water (45) due to the higher rough surface area and the highly ordered structure, resulting in decreasing in modulus.

#### Confocal Laser Scanning Microscopy Analysis

**FIGURE 10:**
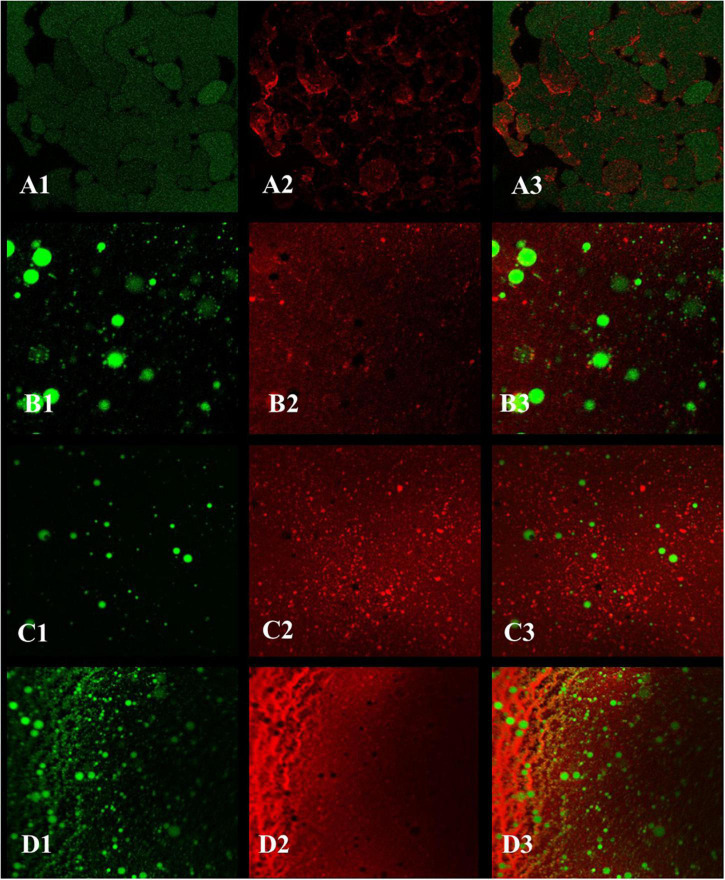
The confocal laser scanning microscopy (CLSM) images of Pickering emulsions [**(A1–A3)** emulsion stabilized by ZCPs; **(B1–B3)** emulsion stabilized by ZLCPs; **(C1–C3)** emulsion stabilized by ZKCPs; **(D1–D3)** emulsion stabilized by ZLKCPs].

For the emulsions prepared by ZCPs (Figures 10A1–A3), ZLCPs (Figures 10B1–B3), ZKCPs (Figures 10C1–C3), and ZLKCPs (Figures 10D1–D3), the green oil droplets covered by protein colloid particles were round and filled in the water phase. As shown in Figures 10A1–A3, the protein formed a specific network structure in the water phase that was conducive to the stability of the emulsion. However, the oil droplets in the emulsion were large, unevenly distributed, and loose, indicating that the emulsion’s stability needed further improvement. With the addition of Lys and KGM, the oil droplets became smaller and more densely packed in the water-phase colloidal particles. As reported by previous studies, the oil droplets individually distributed in all the emulsions and the covering layer could be observed (46). Interaction between the oil droplets formed the gel network. Because the protein coverage and the oil droplets were closely connected, the movement of the droplets and the fluidity of the emulsion were restricted due to the network structure, resulting in a solid-like gel. The oil droplets became even smaller and the network structure became looser with the addition of Lys combined with KGM (Figures 10D1–D3), which may be ascribed to the fact that Lys disturbed the intermolecular forces of zein colloidal particles leading to protein unfolding. The network and the solid-like layer are a barrier to preventing aggregates aggregation of droplets (47). Therefore, adding Lys combined with KGM could decrease emulsion droplets’ size and significantly improve the Pickering emulsion stabilized by ZCPs.

## Conclusion

Emulsions stabilized by ZLKCPs showed the best stability due to the synergistic effects of Lys and KGM. Lys could significantly reduce the average particle size, PDI, and interfacial tension. KGM underwent deacetylation and gelation in Lys solution, decreasing viscosity and modulus. Furthermore, KGM formed a three-dimensional network structure through intramolecular and intermolecular hydrogen bonds due to deacetylation, thereby increasing the stability of Pickering emulsions. Therefore, the interaction between ZCPs, Lys, and KGM effectively promotes emulsions’ stability, in which reaction sequence and reaction time play a crucial role.

## Data Availability Statement

The original contributions presented in this study are included in the article/Supplementary Material, further inquiries can be directed to the corresponding authors.

## Author Contributions

TSo: data curation, formal analysis, resources, methodology, and writing—original manuscript. HL: software, writing review, formal analysis, and editing. AM: software and editing. TSh: software, methodology, and editing. LY: conceptualization, supervision, and editing. RG: funding acquisition, project administration, methodology, and writing—review and editing. All authors contributed to the article and approved the submitted version.

## Conflict of Interest

The authors declare that the research was conducted in the absence of any commercial or financial relationships that could be construed as a potential conflict of interest.

## Publisher’s Note

All claims expressed in this article are solely those of the authors and do not necessarily represent those of their affiliated organizations, or those of the publisher, the editors and the reviewers. Any product that may be evaluated in this article, or claim that may be made by its manufacturer, is not guaranteed or endorsed by the publisher.

## Supplementary Material

Supplementary Figure 1 The appearance **(A)** and centrifugal stability **(B)** of emulsion with ZCPs, L-Lys, and 0 h reaction time.

Supplementary Figure 2 Contact Angle of emulsion stabilized by ZLKCPs.
