# Supplementary figures and images for: Improvement of Storage Stability of Zein-Based Pickering Emulsions by the Combination of Konjac Glucomannan and L-Lysine
Source: Front Nutr. 2022 Jul 11;9:955272. doi: 10.3389/fnut.2022.955272 (PMC9309815; doi:10.3389/fnut.2022.955272)

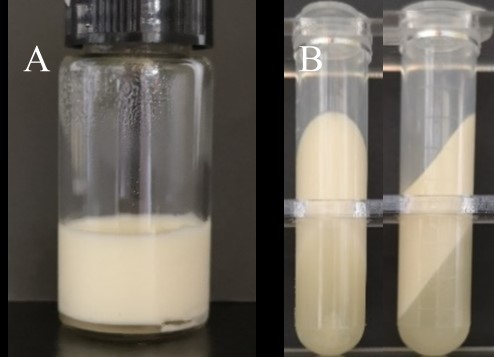

Supplement: Supplementary Figure 1 — The appearance (A) and centrifugal stability (B) of emulsion with ZCPs, L-Lys, and 0 h reaction time. [file Image_1.JPEG]

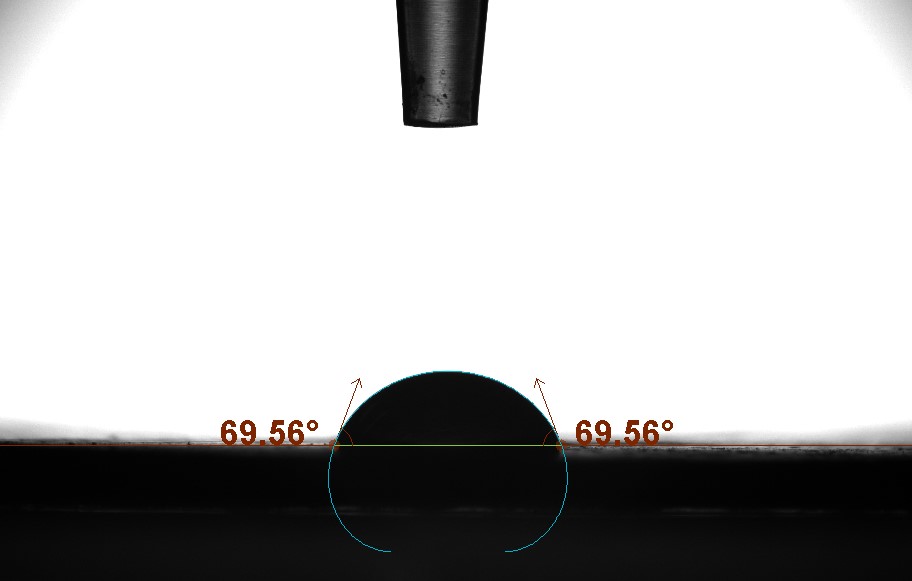

Supplement: Supplementary Figure 2 — Contact Angle of emulsion stabilized by ZLKCPs. [file Image_2.JPEG]
